# Angiogenic cytokines can reflect the synovitis severity and treatment response to biologics in rheumatoid arthritis

**DOI:** 10.1038/s12276-020-0443-8

**Published:** 2020-05-27

**Authors:** Ji‐Won Kim, Jin-Sun Kong, Saseong Lee, Seung-Ah Yoo, Jung Hee Koh, Jingchun Jin, Wan-Uk Kim

**Affiliations:** 1 Division of Rheumatology, Department of Internal Medicine, Catholic University of Daegu School of Medicine, Daegu, Korea; 2 Department of Biomedicine and Health Sciences, The Catholic University of Korea, Seoul, Korea; 3 Center for Integrative Rheumatoid Transcriptomics and Dynamics, The Catholic University of Korea, Seoul, Korea; 4 Division of Rheumatology, Department of Internal Medicine, The Catholic University of Korea, Seoul, Korea; 5 Department of Immunology of Yanbian University Hospital, 133000 Yanji, Jilin Province China; 6 Key Laboratory of Science and Technology Department (Jilin Province), Cancer Research Center, 133002 Yanji, Jilin Province China

**Keywords:** Rheumatoid arthritis, Diagnostic markers, Autoimmune diseases

## Abstract

Angiogenesis and synoviocyte hyperplasia, called ‘pannus,’ are pathologic hallmarks of rheumatoid arthritis (RA). To determine the clinical significance of angiogenic cytokines in RA, the levels of pro-angiogenic cytokines, including VEGF, placenta growth factor (PlGF), and IL-6, were measured in the synovial fluid (SF, *n* = 54) and sera of RA patients (*n* = 157) using ELISA. Patients (*n* = 103) with disease activity score 28 (DAS28) > 3.2, which indicates moderate to high RA activity, underwent follow-up blood sampling at 6 months after treatment with conventional disease-modifying anti-rheumatic drugs (c-DMARD) or biologic DMARD (b-DMARD) including an anti-TNFα antibody, an anti-IL-6 antibody, and abatacept. Ultrasonography (US) was performed on affected joints to define the synovitis severity at the time of sampling. Consequently, in the SF of RA patients, PlGF and IL-6 levels correlated well with synovitis severity determined by US. In RA sera, VEGF and IL-6 levels were elevated in proportion to synovitis severity, correlating with conventional markers for disease activity, including ESR, CRP, and DAS28. In c-DMARD users (*n* = 53), serially monitored levels of serum VEGF, IL-6, erythrocyte sedimentation rate (ESR), and C-reactive protein (CRP) all decreased in good and moderate responders but not in nonresponders. In b-DMARD users (*n* = 49), only serum VEGF well represented the treatment response, while CRP nonspecifically decreased irrespective of the treatment outcome. By multivariable analysis, serum ΔVEGF, but not ΔESR or ΔCRP, was an independent factor associated with good and moderate responses to DMARD. In summary, the angiogenic cytokines PlGF and VEGF represent the synovitis severity of RA assessed by US. In patients receiving b-DMARD, serum VEGF may be more valuable than CRP in reflecting the treatment response.

## Rheumatoid arthritis: growth factor shows diagnostic potential

A growth factor implicated in the development of new blood vessels could serve as an indicator of disease severity and treatment response in people with rheumatoid arthritis (RA). A team led by Wan-Uk Kim from the Catholic University of Korea, Seoul, South Korea, and Jingchun Jin from Yanbian University Hospital, Yanji, China, tested RA patients for signaling proteins that direct the formation of blood vessels. They found several proteins whose levels, either in blood or joint fluid, tracked closely with the severity of joint inflammation. The blood levels of only one protein, vascular endothelial growth factor (VEGF), reliably dropped in those who responded favorably to drug treatments but not in those who derived little benefit from anti-rheumatic therapy. The findings highlight the diagnostic potential of blood testing for VEGF as a biomarker of RA activity.

## Introduction

Rheumatoid arthritis (RA) is pathologically characterized by the abnormal proliferation of synoviocytes and extensive angiogenesis, called pannus formation^1^. In particular, as a major component of the invasive pannus, angiogenesis is believed to be an essential step for the abnormal proliferation of synoviocytes and perpetuation of chronic inflammation by supplying oxygen and nutrients to the tissue and by recruiting immune cells to inflamed sites^2^, respectively. In RA, increased angiogenesis is tightly linked to hypoxic conditions within joints^3–7^. Angiogenesis is mediated by a number of proangiogenic factors, including growth factors, cytokines, and adhesion molecules. A blockade of angiogenesis can ameliorate the development of experimental arthritis in mice, indicating that such pathologic processes can be a therapeutic target for RA^8^.

Vascular endothelial growth factor (VEGF), a representative angiogenic cytokine, can strongly induce endothelial cell activation and proliferation^9,10^. VEGF and its receptors are highly expressed in RA synovial tissues. Their expression levels parallel the degree of synovial angiogenesis^11^. VEGF concentrations are also increased in both the synovial fluid (SF) and serum of RA patients^12–16^. Interestingly, serum VEGF levels are correlated with inflammatory parameters for the disease activity of RA, including erythrocyte sedimentation rate (ESR), C-reactive protein (CRP), and disease activity score 28 (DAS28)^14–18^. We have previously demonstrated that VEGF_165_ confers RA synoviocyte apoptotic resistance^19^, providing important implications for the abnormal growth of synoviocytes, called pannus formation.

Placenta growth factor (PlGF) is a homolog of VEGF that can enhance an angiogenic switch in diseases. PlGF is expressed in RA synovium and SF at high levels^20,21^. It is primarily secreted from RA synoviocytes upon stimulation with pro-inflammatory cytokines^21^. In addition to its role in neovascularization, PlGF can directly increase interleukin (IL)-6 and tumor necrosis factor (TNF)-α production by RA monocytes after binding to its receptor FMS-like tyrosine kinase (Flt)-1. PlGF also promotes proliferation, survival, migration, and invasion of RA synoviocytes^22^, suggesting its role in the formation of the invasive pannus. We have recently demonstrated that PlGF secreted from T_H_17 cells not only increases angiogenesis but also shifts naive T cells toward T_H_17 cell polarization, thereby critically contributing to RA pathogenesis^23^. Together, PlGF shows pleotropic actions for RA pathology possibly by inducing angiogenesis, synoviocyte proliferation, and T_H_17 cell generation.

The paradigm of RA treatment has changed with the introduction of biologic disease-modifying anti-rheumatic drugs (b-DMARD) and, more recently, targeted synthetic DMARD (ts-DMARD). However, treatment responses to b-DMARD vary among patients. Thus, effective biomarkers that can reflect the treatment response are needed. ESR and CRP are blood markers that are currently used. However, they are not always reliable, particularly in patients receiving tocilizumab^24,25^. Therefore, there are unmet needs for alternative biomarkers that can adequately represent disease activity, synovitis severity, and therapeutic responses. Given that fibrovascular hyperplasia called ‘pannus’ is a pathologic hallmark of RA, pannus-related molecules could be reliable biomarkers to address such unmet needs. In this study, we postulated that proangiogenic proteins secreted from synoviocytes could be surrogate biomarkers representing disease activity and severity of RA. To this end, we measured the concentrations of VEGF, PlGF, soluble Flt-1 (sFlt-1), and IL-6 in the SF and/or sera of RA patients and compared them with synovitis severity defined by ultrasonography (US) and therapeutic responses to conventional DMARD (c-DMARD) versus b-DMARD.

## Patients and methods

### Study population and SF and serum sampling

The study population and treatment regimens for patients are summarized in Supplementary Fig. 1. RA patients who fulfilled the 2010 ACR/EULAR classification criteria^26^ and osteoarthritis (OA) controls were recruited from August 2015 to December 2018. SF samples were obtained from RA patients (*n* = 54) and OA controls (*n* = 30) who underwent arthrocentesis on their swollen joints (Supplementary Table 1). Serum samples were also obtained from RA patients (*n* = 157) and OA controls (*n* = 50) at baseline (Supplementary Table 2). Among the 157 RA patients, those with moderate or high disease activity (*n* = 103, DAS28 > 3.2) underwent follow-up serum sampling at 6 months after treatment with c-DMARD (*n* = 53), b-DMARD (*n* = 49), or ts-DMARD (*n* = 1), which was randomly assigned (Supplementary Table 2). This study was approved by the Institutional Review Board of Catholic Medical Center, the Catholic University of Korea (approval number: KC14TIMI0697). Written informed consent was obtained from all study participants.

### VEGF, PlGF, sFlt-1, and IL-6 measurements

VEGF, PlGF, and IL-6 concentrations, which are major proangiogenic factors primarily secreted from proliferating synoviocytes^10,21,27^, were measured from the SF and sera of both RA and OA patients using an enzyme-linked immunosorbent assay (ELISA) as described previously^23^. As a control, the levels of sFlt-1, an anti-angiogenic molecule produced by synoviocytes^23^, were also determined in the same samples by ELISA. The detection limit of ELISA was 10 pg/ml for VEGF, PlGF, IL-6, and sFlt-1.

### Sonographic evaluation of joints for synovitis severity

Musculoskeletal US was performed for all RA patients (*n* = 54) whose SF samples were obtained. In addition, US was also carried out for multiple joints involved (including proximal interphalangeal joints, metacarpophalangeal joints, and wrists) at the time of blood sampling in RA patients (*n* = 68). The synovitis score was determined by gray-scale US (GSUS) and power-Doppler US (PDUS) images (Supplementary Fig. 2). GSUS scores (range, 0–3) were defined by the degree of synovial hypertrophy of the joints as follows: grade 0 = no synovial hypertrophy, grade 1 = minimal synovial hypertrophy, grade 2 = moderate synovial hypertrophy, and grade 3 = evere synovial hypertrophy. PDUS scores (range, 0–3) were assessed by the extent of vascularity within the synovium of joints as follows: grade 0 = no Doppler activity, grade 1 = minimal Doppler activity, grade 2 = moderate Doppler activity (<50% of the background synovium), and grade 3 = severe Doppler activity (>50% of the background synovium)^28,29^. Patients were considered to have active synovitis when GSUS was ≥2 or PDUS was ≥1.

### Assessment of clinical parameters

The tender joint count (TJC), swollen joint count (SJC), ESR level, and CRP level of RA patients were examined at baseline and follow-up. Data on serologic markers, including rheumatoid factor (RF) and anti-citrullinated peptide antibodies (ACPA), were obtained. Radiographic examination of hands and feet was performed. The degree of joint space narrowing and erosion was scored according to the van der Heijde-modified Sharp score (vdH Sharp score)^30^. The disease activity of RA patients was determined based on the disease activity score 28_ESR_ (DAS28_ESR_) as follows: DAS28 ≤ 3.2 = low disease activity; DAS28 > 3.2 = moderate or high disease activity^31^. European League Against Rheumatism (EULAR) response criteria were employed to assess the treatment response^32^.

### Statistical analysis

Comparison of continuous variables was examined by Student’s unpaired *t* test or Mann–Whitney *U* test. Comparison of categorical variables was performed using the Chi-square test or Fisher’s exact test. Correlations between two variables were analyzed using Pearson’s correlation test. A paired *t* test was employed to examine the difference between baseline and follow-up values of the same subject. Simple and multiple logistic regression analyses were performed to investigate factors associated with a good or moderate response to the treatment. Multiple logistic regression was performed for variables with *p* values less than 0.05 in simple logistic regression. All statistical analyses were conducted using IBM SPSS Statistics 20 (IBM Corp., Armonk, NY, USA). Graphs were drawn using GraphPad Prism 5 (GraphPad Software, San Diego, CA, USA). Heatmap correlation images were drawn using the package ‘corrplot’ from R software 3.5.1 (R Project, Vienna, Austria).

## Results

### Angiogenic cytokine PlGF and IL-6 levels in the SF correlate with synovitis severity and systemic inflammatory response in RA

**Fig. 1:**
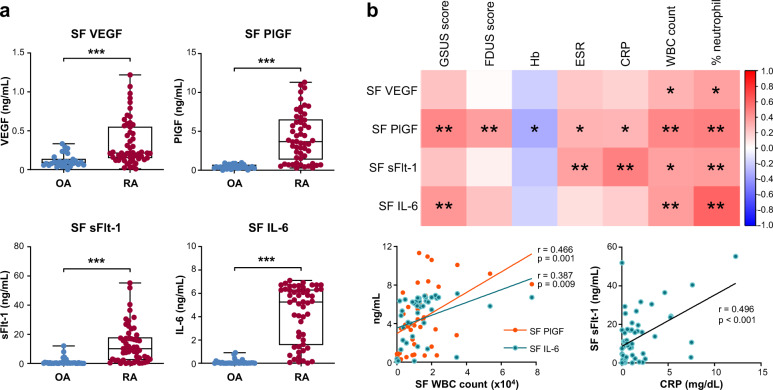
Expression of angiogenic factors in the synovial fluids (SF) and their association with local and systemic inflammatory responses in rheumatoid arthritis (RA) patients. **a** Vascular endothelial growth factor (VEGF), placenta growth factor (PlGF), soluble Flt-1 (sFlt-1), and interleukin (IL)-6 concentrations in the SF of RA patients (*n* = 54) and osteoarthritis (OA) controls (*n* = 30). Bars indicate the mean and SD. **b** Correlations of VEGF, PlGF, sFlt-1, and IL-6 in the SF with synovitis severity on ultrasonography (US), hemoglobin, erythrocyte sedimentation rate (ESR), C-reactive protein (CRP), white blood cell (WBC) count, and % neutrophils in the SF of RA patients. A correlation matrix heatmap is presented in the upper panel, and correlation plots are shown in the lower panel. GSUS: gray-scale US; PDUS: power-Doppler US. *p < 0.05, **p < 0.01, ***p < 0.001 by Student’s unpaired *t* test or Pearson’s correlation *t*est.

As reported previously^12,21,33^, the concentrations of VEGF, PlGF, sFlt-1, and IL-6 were significantly higher in the SF of RA patients than in that of OA controls (Fig. 1a). We then tested whether the levels of VEGF, PlGF, and IL-6, as pro-angiogenic cytokines mainly secreted from synoviocytes, could represent local and/or systemic inflammatory responses in RA patients. As shown in Fig. 1b and Supplementary Table 1, PlGF concentrations in the SF (*n* = 54) were well correlated with WBC counts (*r* = 0.466 and *p* = 0.001) and % neutrophils in the SF, as well as with blood levels of ESR and CRP as markers for systemic inflammatory responses in RA. Levels of sFlt-1, an anti-angiogenic protein used as a control^23^, also had a positive relationship with inflammatory markers in the blood (*r* = 0.364 and *p* = 0.007 for ESR, *r* = 0.496 and *p* < 0.001 for CRP). However, VEGF and IL-6 levels in the SF failed to correlate with ESR and CRP, although they showed positive correlations with WBC counts and % neutrophils in the SF (Fig. 1b).

**Fig. 2:**
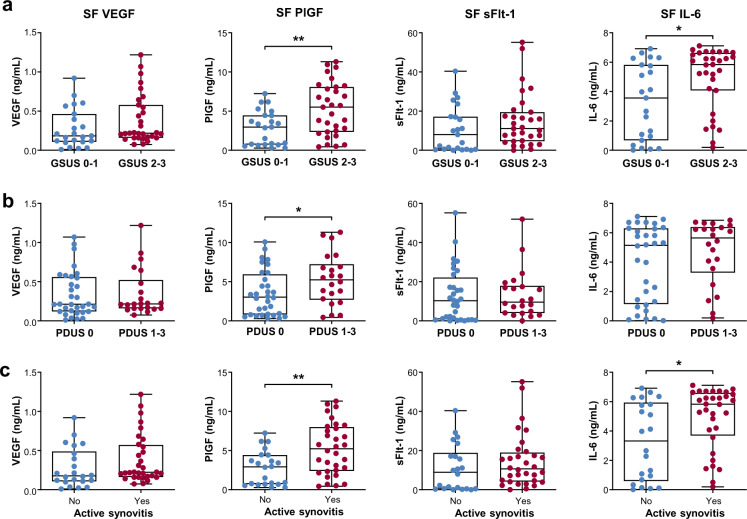
Levels of VEGF, PlGF, sFlt-1, and IL-6 in the SF according to synovitis severity on US. **a** VEGF, PlGF, sFlt-1, and IL-6 concentrations in the SF of RA patients with significant synovial hypertrophy (gray-scale US, GSUS 2 and 3) versus those with no or mild synovial hypertrophy (GSUS 0 and 1). **b** VEGF, PlGF, sFlt-1, and IL-6 concentrations in RA patients with increased vascularity (power-Doppler US, PDUS 1 to 3) and in those without (PDUS 0). **c** VEGF, PlGF, sFlt-1, and IL-6 concentrations in RA patients with active synovitis and in those without. Active synovitis was defined as GSUS ≥ 2 or PDUS ≥ 1. GSUS: gray-scale US; PDUS: power-Doppler US. Bars indicate the mean and SD. *p < 0.05, **p < 0.01 by Student’s unpaired *t* test.

We next questioned whether angiogenic cytokines could reflect the synovitis severity determined by US in RA patients. The results showed that PlGF and IL-6 concentrations in the SF of patients with moderate to severe synovial hypertrophy on GSUS (grades 2 and 3) were higher than those in the SF of patients with no or mild synovial hypertrophy on GSUS (grades 0 and 1) (Fig. 2a). On PDUS examination, the PlGF concentration was also greater in patients with increased vascularity (grade 1 to 3) than in those without (grade 0) (Fig. 2b), whereas the other three molecules did not show significant differences according to the presence of increased vascularity. When we divided patients into two groups depending on the presence of active synovitis (GSUS ≥ 2 or PDUS ≥ 1), the levels of PlGF and IL-6, but not VEGF or sFlt-1, were significantly higher in patients with active synovitis than in those with inactive synovitis (Fig. 2c).

Taken together, these results demonstrate that the levels of PlGF and IL-6, as pro-angiogenic factors primarily produced by proliferating synoviocytes, are elevated in the SF of RA patients. Such levels can represent the synovitis severity, as well as the local and systemic inflammatory status of RA patients.

### Circulating levels of VEGF and IL-6, but not PlGF, correlate with disease activity and severity of RA

**Fig. 3:**
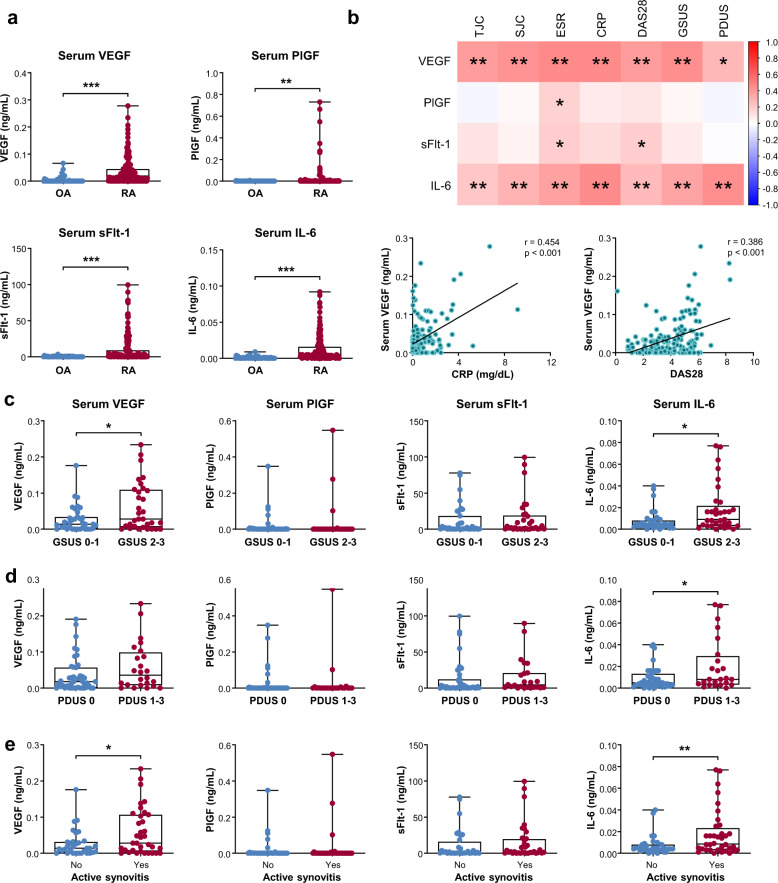
Levels of VEGF, PlGF, sFlt-1, and IL-6 in the sera according to sonographic synovitis severity and disease activity of RA. **a** VEGF, PlGF, sFlt-1, and IL-6 concentrations in the sera of RA patients (*n* = 157) and OA controls (*n* = 50). **b** Correlations of serum VEGF, PlGF, sFlt-1, and IL-6 levels with tender joint count, swollen joint count, ESR, CRP, disease activity score 28 (DAS28), and US score of synovitis. The correlation matrix heatmap and correlation plots are shown in the upper panel and the lower panel, respectively. **c**, **d**, and **e** Serum concentrations of VEGF, PlGF, sFlt-1, and IL-6 depending on the severity of synovial hypertrophy (**c**), the extent of vascularity (**d**), or the presence of active synovitis (**e**). Active synovitis was defined as GSUS ≥ 2 or PDUS ≥ 1. Bars indicate the mean and SD. *p < 0.05, **p < 0.01, ***p < 0.001 by Student’s unpaired *t* test or Pearson’s correlation test.

Serum samples were obtained from 157 RA patients (54 patients with low disease activity and 103 patients with moderate or high disease activity). The baseline demographic and disease characteristics of these patients are summarized in Supplementary Table 2. We first confirmed that serum VEGF, PlGF, sFlt-1, and IL-6 concentrations were increased in RA patients (Fig. 3a). As expected, in comparison with parameters for RA disease activity, VEGF and IL-6 levels were correlated with TJC, SJC, ESR, CRP, and DAS28 (Fig. 3b), consistent with earlier reports^16–18,34,35^. In addition, as shown in the heat map and correlation plots in Fig. 3b, serum VEGF and IL-6 levels were positively correlated with both GSUS and PDUS scores. Moreover, serum VEGF and IL-6 concentrations were higher in patients with moderate to severe synovial hypertrophy on GSUS (Fig. 3c) and in patients with increased vascularity on PDUS than in those without (Fig. 3d). Moreover, these higher concentrations were significantly associated with the presence of active synovitis (Fig. 3e).

In sharp contrast with the SF data, the serum PlGF level did not correlate with TJC, SJC, CRP, or DAS28 (Fig. 3b). No significant difference in serum PlGF concentration was found according to sonographic severity. Serum levels of sFlt-1, an anti-angiogenic protein^23^, showed only modest correlations with ESR and DAS28 and failed to show any relationship with TJC, SJC, CRP, or synovitis severity on GSUS or PDUS.

Collectively, these results indicate that serum levels of VEGF and IL-6, but not serum PlGF or serum sFlt-1, could represent synovial proliferation and hypervascularity on US and reflect the systemic inflammatory status of RA assessed by TJC, SJC, ESR, CRP, and DAS28.

### Serum VEGF is better at reflecting the treatment response to b-DMARD than ESR or CRP

We next investigated whether the serum angiogenic factors VEGF and IL-6 could be used as indicators of the treatment response since they correlated well with the disease activity of RA. To this end, serum VEGF and IL-6, as well as ESR and CRP were serially monitored in active RA patients whose DAS28 score was >3.2 at study entry and then compared with EULAR response criteria. The baseline characteristics of c-DMARD users (*n* = 53) and b-DMARD users (*n* = 49) are described in Supplementary Table 3. One patient treated with ts-DMARD was excluded from the analysis. Age, sex, BMI, and autoantibody status were similar between the two groups. However, ESR and DAS28 at baseline were higher in b-DMARD users than in c-DMARD users.

**Fig. 4:**
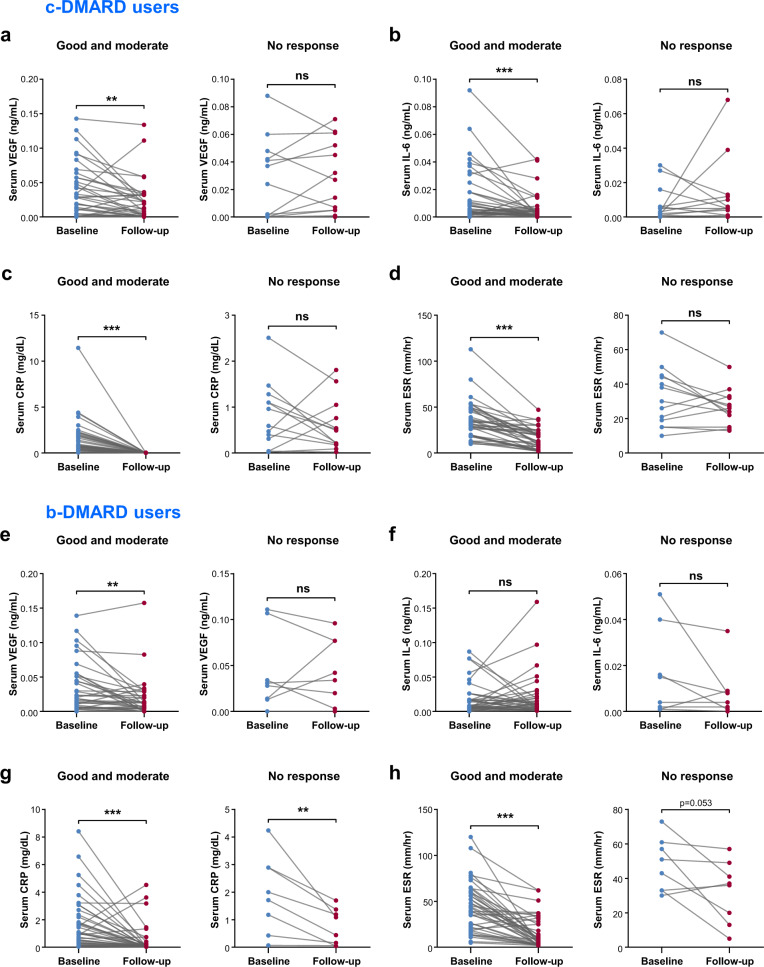
Serial monitoring of serum VEGF, IL-6, CRP, and ESR levels at baseline and at 6 months after treatment with conventional DMARD (c-DMARD) versus biologic DMARD (b-DMARD). **a**–**d** Changes in serum levels of VEGF, IL-6, CRP, and ESR in c-DMARD users (*n* = 53). The left panel shows good or moderate responders (*n* = 40), and the right panel shows nonresponders (*n* = 13). **e**–**h** Changes in serum levels of VEGF, IL-6, CRP, and ESR in b-DMARD users (*n* = 49). The left panel shows good or moderate responders (*n* = 41), and the right panel shows nonresponders (*n* = 8). EULAR response criteria were used to define good responders, moderate responders, and nonresponders in the two groups. Ns = not significant, **p < 0.01, ***p < 0.001 by the paired *t* test.

As a result, in good and moderate responders with c-DMARD, serum VEGF and IL-6, as well as ESR and CRP significantly decreased after 6 months of treatment (Fig. 4a–c, and d) but were not altered in nonresponders. These data indicate that changes in the pro-angiogenic cytokines VEGF and IL-6, in addition to ESR and CRP, could well reflect the treatment response to c-DMARD, thus discriminating the responder group from the nonresponder group. In support of this finding, ΔDAS28 was positively correlated with ΔESR and ΔCRP, as well as with ΔVEGF and ΔIL-6 (Supplementary Fig. 3a).

In b-DMARD users, serum VEGF levels significantly decreased in good or moderate responders and showed no significant change in nonresponders, similar to the results in c-DMARD users (Fig. 4e). However, serum IL-6 levels were not significantly changed in responders or nonresponders (Fig. 4f), indicating no role of IL-6 in reflecting the treatment response to b-DMARD. This absent role of IL-6 seemed to be associated with the use of tocilizumab, an antibody against the IL-6 receptor, because subgroup analysis demonstrated that ΔIL-6 showed no association with ΔDAS28 in patients treated with tocilizumab but not in those treated with non-tocilizumab biologics (Supplementary Fig. 3b, c, d). Interestingly, although CRP levels considerably decreased in responders as expected, they were also reduced in nonresponders after b-DMARD treatment (Fig. 4g). The ESR level tended to show a similar pattern (Fig. 4h).

Taken together, our data suggest that, in contrast with the c-DMARD subgroup, in patients receiving b-DMARD, CRP can be nonspecifically decreased irrespective of the therapeutic response and that ΔVEGF might be a good alternative to assess the treatment response in a specific subgroup of biologics users.

### Change in serum VEGF level is an independent factor associated with a good and moderate response to DMARD

Based on our finding that serum VEGF, but not CRP, well represented the disease activity and treatment response in both c-DMARD- receiving and b-DMARD-receiving patients, we further investigated whether VEGF could serve as an independent indicator of the therapeutic response. Baseline characteristics of good/moderate responders (*n* = 82) and nonresponders (*n* = 21) are described in Supplementary Table 4. The proportions of cDMARD users and bDMARD users did not significantly differ between good/moderate responders and nonresponders. Baseline VEGF levels were similar between good/moderate responders and nonresponders. However, when laboratory parameters and serum ΔVEGF levels (serum VEGF concentration at baseline – VEGF concentration at 6 months after treatment with DMARD) were compared between the two groups, good/moderate responders had older age, higher ΔESR, and higher ΔVEGF than nonresponders (Supplementary Table 4). These data suggest that these parameters, including changes in VEGF levels, could serve as surrogate biomarkers to reflect the treatment response to DMARD.

Multivariable logistic regression analysis was performed to assess whether serum ΔVEGF could independently reflect the treatment response to DMARD. The multivariable logistic model consisted of age, ΔESR/ΔCRP, and ΔVEGF, which showed statistical significance in the univariable analysis. As a result, ΔVEGF was found to be an independent factor associated with a good/moderate response to DMARD with an odds ratio of 3.55. Of note, ΔESR and ΔCRP levels failed to show a significant predictive power (Table 1), consistent with our finding of a nonspecific reduction of CRP by b-DMARD irrespective of clinical outcome (Fig. 4g, h).

**Table 1:** Factors associated with good and moderate response in RA patients.

Variable	Univariable		Multivariable	
	Unadjusted OR	*p*	Adjusted OR	*p*
Age	**1.05 (1.01–1.10)**	**0.026**	1.05 (0.99–1.12)	0.056
BMI ≥ 25	1.46 (0.44–4.84)	0.533		
Duration of disease, years	1.02 (0.96–1.08)	0.537		
Abnormal ESR	0.60 (0.18–1.99)	0.406		
Abnormal CRP	1.02 (0.37–2.82)	0.972		
Baseline DAS28	1.60 (0.93–2.74)	0.088		
High-positive RF	0.87 (0.32–2.39)	0.782		
High-positive ACPA	0.34 (0.07–1.59)	0.170		
vdH Sharp score	0.99 (0.98–1.00)	0.132		
Glucocorticoid use	2.48 (0.93–6.65)	0.071		
b-DMARD use	1.63 (0.61–4.34)	0.332		
Abatacept use	0.58 (0.13–2.65)	0.480		
Tocilizumab use	2.13 (0.38–11.83)	0.389		
High VEGF	1.95 (0.65–5.84)	0.235		
High IL-6	2.33 (0.87–6.23)	0.091		
**High ΔVEGF (baseline-F/U)**	**4.67 (1.70–12.82)**	**0.003**	**3.55 (1.15–10.93)**	**0.027**
High ΔIL-6 (baseline-F/U)	1.88 (0.71–5.02)	0.207		
High ΔESR (baseline-F/U)	**4.21 (1.53–11.54)**	**0.005**	2.27 (0.72–7.10)	0.160
High ΔCRP (baseline-F/U)	**8.93 (1.14–70.26)**	**0.038**	5.76 (0.68–48.62)	0.108

Variables with *p* values less than 0.05 were entered into multivariable logistic regression analysis.

*OR* odds ratio, *BMI* body mass index, *ESR* erythrocyte sedimentation rate, *CRP* C-reactive protein, *DAS28* disease activity score 28, *RF* rheumatoid factor, *ACPA* anti-citrullinated peptide antibodies, *vdH* van der Heijde, *bDMARD* biologic disease-modifying anti-rheumatic drug, *VEGF* vascular endothelial growth factor; *IL-6* interleukin-6, and *F/U* follow-up.

Values in bold indicate statistical significance (*p* < 0.05).

## Discussion

In this study, we postulate that angiogenic cytokines secreted from RA synoviocytes could specifically represent a ‘pannus’ pathology and well reflect the pathologic and inflammatory status of RA when they overflow into the periphery. We tested whether the levels of the angiogenic cytokines VEGF, IL-6, and PlGF, as major pro-angiogenic cytokines in RA, could reflect the synovitis severity, disease activity, and treatment response in RA patients. We demonstrated that the expression levels of PlGF and VEGF were elevated in proportion to synovitis severity as assessed by GSUS and/or PDUS. In c-DMARD users, serially monitored serum VEGF, IL-6, ESR, and CRP levels all decreased in good and moderate responders but were not significantly changed in nonresponders. In contrast, in b-DMARD users, only changes in serum VEGF well represented the treatment response, having a better value than ESR and CRP. Importantly, changes in serum VEGF were an independent factor associated with good and moderate responses. Taken together, our data suggest that PlGF and VEGF, as angiogenic cytokines, are correlated with synovitis severity and disease activity in RA patients and that serum VEGF has a better value to represent treatment response than ESR and CRP.

Musculoskeletal US has become a useful diagnostic tool in clinical practice and research because it well represents joint pathology without performing invasive methods^36^. US can monitor the clinical outcomes of a therapy. Improvement in the power Doppler grade, along with the improvement in disease activity, has been found after treatment with TNF-α inhibitors^37–39^. In addition, power Doppler grade at baseline may help predict relapse in RA patients who have achieved clinical remission^40–42^. In this study, VEGF, PlGF, and IL-6 were selected for comparison with US findings of joints because they are all pro-angiogenic factors predominantly secreted from hypertrophic synoviocytes. We found that in the synovial compartment, PlGF and IL-6 levels, but not VEGF or sFlt-1, were higher with increasing vascularity on PDUS and synovial hypertrophy on GSUS. Interestingly, in the peripheral compartment, serum VEGF and IL-6 levels increased in proportion to synovitis severity on US, correlating with conventional disease activity markers including ESR, CRP, and DAS28, whereas serum PlGF failed to show such correlation, which is consistent with earlier reports showing that PDUS grade correlates with serum VEGF^43,44^. It is unclear why serum PlGF did not reflect RA activity, but this finding may be associated with the very low level of PlGF in RA sera in contrast to that in RA SF; indeed, serum PlGF was detectable (above 10 pg/ml) in only 18 (11.5%) out of 157 RA patients tested, whereas serum VEGF was measurable in the majority of the RA patients (100 patients, 63.7%). Considering that fibrovascular hyperplasia is a characteristic pathology of RA, these results suggest that the proangiogenic cytokines PlGF and VEGF can be used as surrogate markers for synovial pathology, although their clinical roles are different depending on body compartment.

One of the major goals of RA treatment is to maintain minimal residual disease or complete remission^45^. The blood markers ESR and CRP have been widely used for RA assessment. However, they do not always precisely reflect the disease activity or treatment response^46–49^. For example, ESR can be influenced by anemia and age^46,47^. Importantly, ESR and CRP are nonspecifically elevated by infection, and they remain normal in more than a third of RA patients at presentation irrespective of disease activity^48,49^. Moreover, CRP may not be a reliable biomarker in patients with anti-IL-6 blocking agents because IL-6 is crucial to the hepatic biosynthesis of CRP^48,49^. Although DAS28 has been widely used for RA assessment, it is also based on ESR or CRP measurements. In these instances, there is an unmet need for the identification of reliable biomarkers for more precisely assessing the disease activity and treatment response in RA patients, particularly in patients treated with biologics.

In this study, serum VEGF and IL-6 levels were correlated with TJC, SJC, ESR, CRP, and DAS28, consistent with previous studies demonstrating the association of serum VEGF with RA activity measures, including acute phase reactants and DAS28^16–18,34^. Serum VEGF is reduced in RA patients who experience clinical remission after c-DMARD treatment^15^, suggesting that it is an indicator of treatment response. However, it remains to be determined whether changes in serum VEGF levels could discriminate responders from nonresponders to b-DMARD. Moreover, no trial has been conducted to compare the diagnostic performance of the conventional biomarker ESR and CRP between c-DMARD and b-DMARD users. In a prospective trial, we found that in c-DMARD users, serum VEGF, IL-6, CRP, and ESR were all reduced in correlation with the treatment response. In b-DMARD-treated patients, serum VEGF also decreased in good or moderate responders but not in nonresponders, indicating that serum VEGF could help distinguish responders from nonresponders among b-DMARD users. In contrast, ESR and CRP were nonspecifically decreased in b-DMARD users irrespective of the clinical outcome. Serum IL-6 levels were also irrelevant to the responsiveness to b-DMARD. Taken together, these data suggest that serum VEGF could be an alternative biomarker for adequately monitoring the therapeutic response to DMARD and that the diagnostic value of CRP should be readjusted in patients treated with b-DMARD.

Our study population is unique in that it consisted of similar numbers of c-DMARD cases (*n* = 53) and b-DMARD cases (*n* = 49). In a multivariable logistic regression analysis performed in this population, we found that ΔVEGF was an independent factor associated with a good or moderate response to DMARD. Notably, ΔESR and ΔCRP levels failed to show statistical significance in relation to the treatment response. These results, together with our findings of nonspecific reduction of CRP by b-DMARD, suggest that serum VEGF may be better at reflecting the treatment response to DMARD, particularly to b-DMARD, than ESR or CRP. In this regard, the concept of DAS28 using serum VEGF could be introduced. If a new composite score, such as DAR28_VEGF_, is reasonably established, it would be interesting to test its diagnostic performance in comparison to that of DAS28_ESR_ and DAS28_CRP_.

A limitation of this study was that the number of patients for each group of c-DMARD and b-DMARD cases was relatively too small to reach a strong conclusion. Moreover, the study population of the b-DMARD subgroup was heterogeneous, consisting of 19 patients treated with tocilizumab, 10 patients treated with TNF-α inhibitors, 19 patients treated with abatacept, and 1 patient treated with rituximab. Therefore, it was inconclusive which kind of b-DMARD primarily contributed to our conclusion. Further studies on a large scale are required to address this issue. Notwithstanding those limitations, our investigation has strength in that it is the first proof-of concept study demonstrating that angiogenic cytokines PlGF and VEGF are surrogate markers that represent the sonographic severity of synovial pathology. Moreover, this is the first study suggesting that VEGF might be better at representing the treatment response than ESR or CRP, particularly in RA patients treated with b-DMARD.

In summary, in a single-center prospective study, we found that the angiogenic cytokines PlGF and VEGF, which are major pro-angiogenic cytokines secreted from synoviocytes, were elevated in the SF and sera of RA patients and that their expression was increased with increasing synovitis severity. Serum VEGF levels correlated with disease activity and treatment response in both c-DMARD-treated and b-DMARD-treated patients. In contrast, the traditional biomarker CRP was nonspecifically reduced by b-DMARD irrespective of the clinical outcome. On the other hand, a change in serum VEGF was an independent factor representing the therapeutic response to DMARD. Conclusively, our results suggest that the diagnostic value of CRP should be reconsidered in the era of biologics and that serum VEGF could be an alternative to ESR and CRP, which are the two widely accepted biomarkers of RA activity.

## Supplementary information

Supplementary material
