# Supplementary material for: Angiogenic cytokines can reflect the synovitis severity and treatment response to biologics in rheumatoid arthritis
Source: Exp Mol Med. 2020 May 27;52(5):843–53. doi: 10.1038/s12276-020-0443-8 (PMC7272442; doi:10.1038/s12276-020-0443-8)
Supplement: Supplementary file 1 — Supplementary material [file 12276_2020_443_MOESM1_ESM.pdf]

Angiogenic cytokines can reflect synovitis severity and treatment response to biologics in rheumatoid arthritis

Ji-Won Kim[1], Jin-Sun Kong[2,3], Saseong Lee[2,3], Seung-Ah Yoo[2,3], Jung Hee Koh[4], Jingchun Jin[5,6], and Wan-Uk Kim[2,3,4 *]

[1]Division of Rheumatology, Department of Internal Medicine, Catholic University of Daegu School of Medicine, Daegu, Korea·

[2]Department of Biomedicine & Health Sciences, The Catholic University of Korea, Seoul, Korea

[3]Center for Integrative Rheumatoid Transcriptomics and Dynamics, The Catholic University of Korea, Seoul, Korea

[4]Division of Rheumatology, Department of Internal Medicine, The Catholic University of Korea, Seoul, Korea

[5]Department of Immunology of Yanbian University Hospital, Yanji 133000, Jilin Province, China

[6]Key Laboratory of Science and Technology Department (Jilin Province), Cancer Research Center, Yanji 133002, Jilin Province, China

This work was supported by a grant from the National Research Foundation of Korea (NRF) funded by the Ministry of Education, Science and Technology (No. 2015R1A3A2032927 to W.U.K and 2018R1D1A1B07045491 to J.H.K).

Correspondence and reprint requests to:

Dr. Wan-Uk Kim, Division of Rheumatology, Department of Internal Medicine, Catholic University of Korea, School of Medicine, Seoul, Korea (E-mail: wan725@catholic.ac.kr) or Dr. Jingchun Jin, Department of Immunology of Yanbian University Hospital, Yanji 133000, Jilin Province, China (E-mail: jingchun680928@163.com)

Keywords: vascular endothelial growth factor**,** placenta growth factor, ultrasonography, synovitis severity, treatment response

Running title: Clinical role of angiogenic cytokines in RA

Supplementary figure legends

Supplementary Fig. 1. Flow chart describing treatment regimen of RA patients enrolled for serum sampling. DA, disease activity; c-DMARD: conventional disease-modifying anti-rheumatic drug; b-DMARD: biologic disease-modifying anti-rheumatic drug; ts-DMARD: targeted synthetic disease-modifying anti-rheumatic drug; and TNFi: tumor necrosis factor inhibitor.

Supplementary Fig. 2. A representative of ultrasonographic findings demonstrating synovial hypertrophy and hyper-vascularity. (**a** and **b**) Grey scale US images showing synovial hypertrophy in proximal interphalangeal (PIP) joints. (**c** and **d**) Power-Doppler US images showing vascularity of the synovium in PIP joints.

Supplementary Fig. 3. Correlations of △DAS28 with △ESR, △CRP, △VEGF, and △IL-6 levels in patients treated with c-DMARD (a), b-DMARD (b), Tocilizumab (TCZ) (c), or non-TCZ biologics (d).

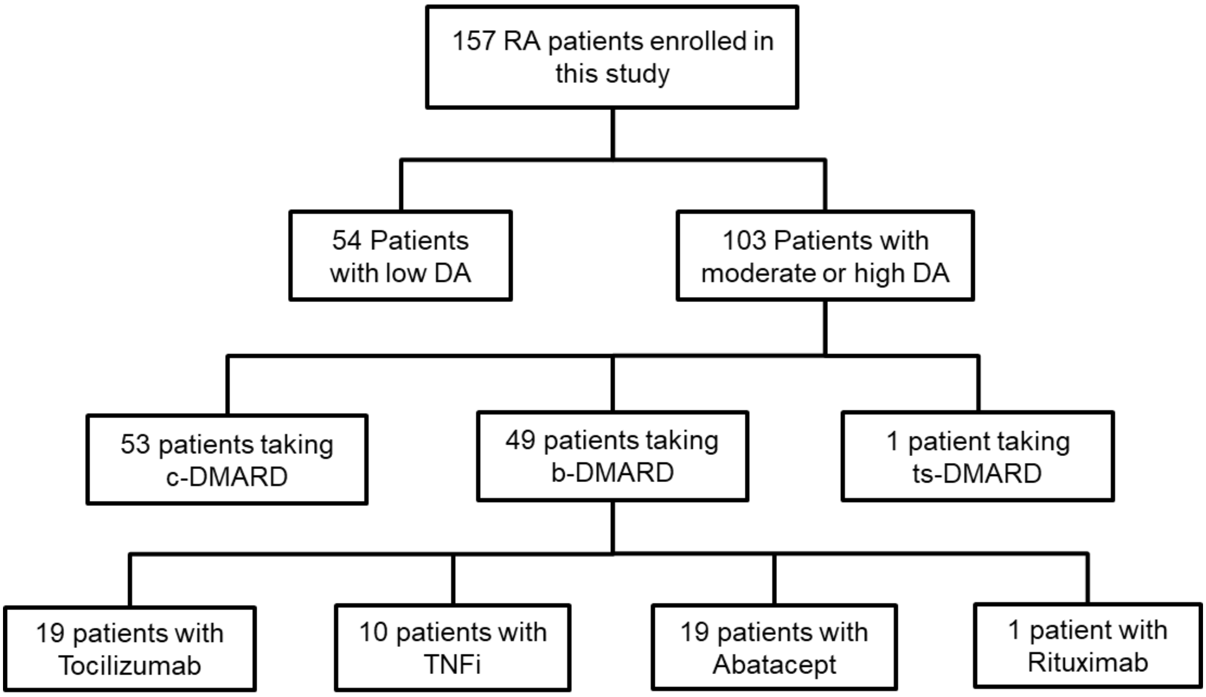

Supplementary Fig. 1. Flow chart describing treatment regimen of RA patients enrolled for serum sampling. DA, disease activity; c-DMARD: conventional disease-modifying anti-rheumatic drug; b-DMARD: biologic disease-modifying anti-rheumatic drug; ts-DMARD: targeted synthetic disease-modifying anti-rheumatic drug; and TNFi: tumor necrosis factor inhibitor.

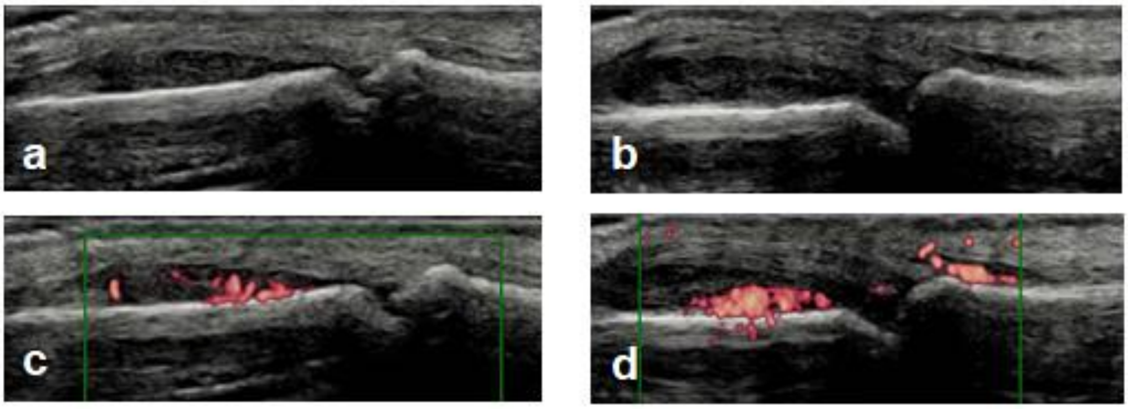

Supplementary Fig. 2. A representative of ultrasonographic findings demonstrating synovial hypertrophy and hyper-vascularity. (**a** and **b**) Grey scale US images showing synovial hypertrophy in proximal interphalangeal (PIP) joints. (**c** and **d**) Power-Doppler US images showing vascularity of the synovium in PIP joints.

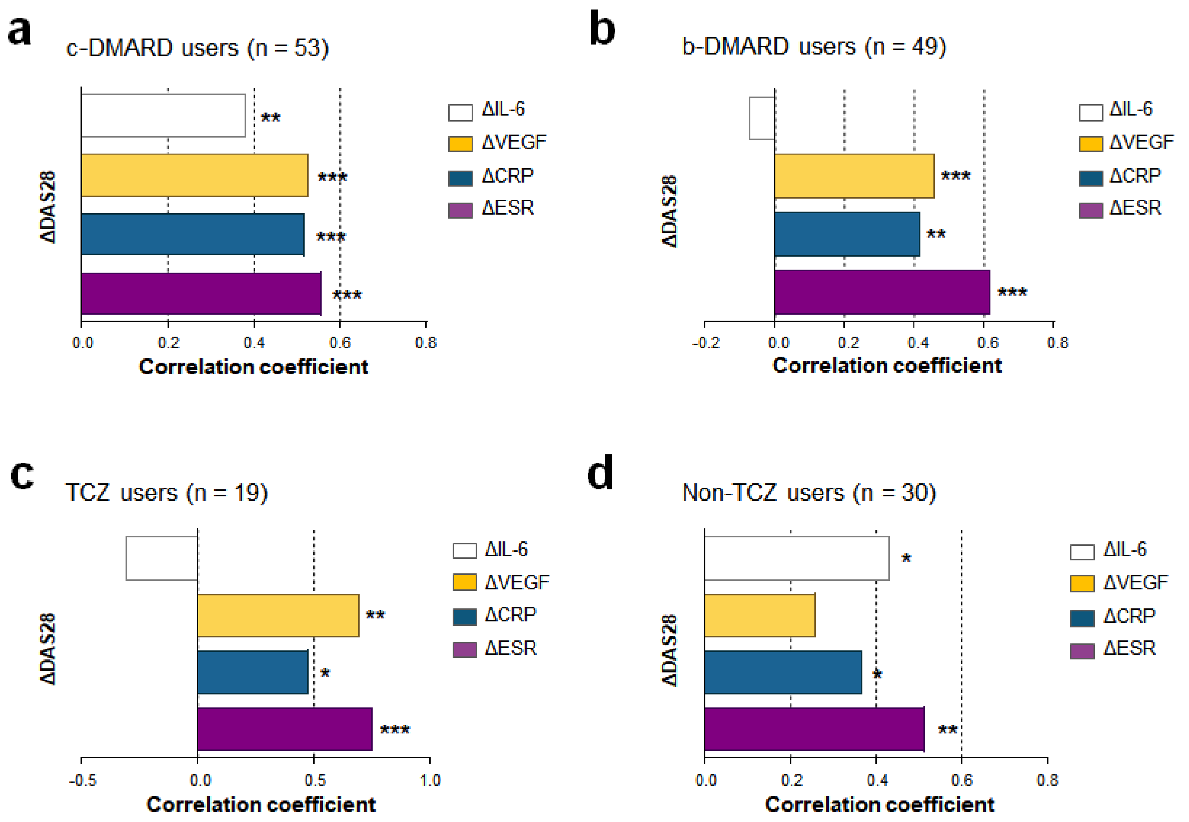

Supplementary Fig. 3. Correlations of △DAS28 with △ESR, △CRP, △VEGF, and △IL-6 levels in patients treated with c-DMARD (a), b-DMARD (b), Tocilizumab (TCZ) (c), or non-TCZ biologics (d).

Supplementary Table 1. Characteristics of RA patients whose synovial fluids were obtained

Variable	Total patients (n=54)
Age, years	59.3 (12.4)
Female, n (%)	51 (94.4)
Serum RF, IU/mL	59.6 (7.5-173.7)
Serum ACPA, U/mL	84.0 (5.4-291.3)
Blood WBC count, /μL	7,390 (6,373-8,958)
Blood % neutrophil	65.1 (10.6)
Hemoglobin, g/dL	12.3 (1.4)
ESR, mm/hour	22.5 (12.8-39.3)
CRP, mg/dL	0.89 (0.20-2.08)
WBC count in the SF, /μL	11,700 (4,275-17,200)
% neutrophil in the SF	63.0 (43.0-75.5)
Glucocorticoid use, n (%)	46 (85.2)
Glucocorticoid dose, PD equivalent, mg/day	5.0 (2.5-7.5)
Methotrexate use, n (%)	28 (51.9)
GSUS grade ≥2, n (%)	31 (57.4)
PDUS grade ≥1, n (%)	22 (40.7)
Active synovitis (GSUS ≥2 or PDUS ≥1), n (%)	32 (59.3)

Data are expressed as mean (standard deviation) or median (interquartile range) for continuous variables. RF: rheumatoid factor; ACPA: anti-citrullinated peptide antibodies; WBC: white blood cell; ESR: erythrocyte sedimentation rate; CRP: C-reactive protein; SF: synovial fluid; PD: prednisone; GSUS: grey scale ultrasound; and PDUS: power-Doppler ultrasound.

Supplementary Table 2. Baseline characteristics of RA patients whose sera were obtained

Variable	Total (n=157)	Low DA (n=54)*	Moderate or High DA (n=103)*	p value*
Age, years	56 (11.6)	55.9 (11.8)	56 (11.6)	0.951
Female, n (%)	136 (86.6)	45 (83.3)	91 (88.3)	0.380
BMI, kg/m^2	23 (20.8-24.4)	22.8 (20.5-23.9)	23.1 (21-24.9)	0.125
Duration of disease, years	5 (1-13.5)	7 (3-12.3)	4 (0-15)	**0.026**
ESR, mm/hour	26 (13-44)	12 (3.8-21)	36 (20-52)	**<0.001**
CRP, mg/dL	0.42 (0.08-1.42)	0.06 (0.02-0.21)	0.90 (0.34-2.0)	**<0.001**
RF, IU/mL	99 (33.7-362.7)	69.7 (19.9-156.2)	121 (40.4-476.7)	**0.015**
ACPA, U/mL	171.3 (37-340)	200 (42.3-340)	163.5 (34.9-340)	0.949
Glucocorticoid use, n (%)	110 (70.1)	39 (72.2)	71 (68.9)	0.669
PD equivalent, mg/day	2.5 (0-5)	2.5 (0-5)	2.5 (0-5)	0.772
Previous c-DMARD use, n (%)	135 (86.0)	51 (94.4)	84 (81.6)	**0.027**
MTX dose, mg/week	7.5 (0-10)	7.5 (0-10)	7.5 (0-10)	0.532
Previous b-DMARD use, n (%)	28 (17.8)	19 (35.2)	9 (8.7)	**<0.001**

Data are expressed as mean (standard deviation) or median (interquartile range) for continuous variables. *Comparison of characteristics between patients with low DA and those with moderate or high DA by student's unpaired *t* test. DA: disease activity; BMI: body mass index; ESR: erythrocyte sedimentation rate; CRP: C-reactive protein; RF: rheumatoid factor; ACPA: anti-citrullinated peptide antibodies; PD: prednisone; c-DMARD: conventional disease-modifying anti-rheumatic drug; MTX: methotrexate; and b-DMARD: biologic disease-modifying anti-rheumatic drug.

Supplementary Table 3. Baseline characteristics of RA patients treated with c-DMARD versus b-DMARD

Variable	c-DMARD users (n=53)	b-DMARD users (n=49)	p value
Age, years	55.9 (11.9)	56.0 (11.5)	0.960
Female, n (%)	46 (86.8)	44 (89.8)	0.638
BMI, kg/m^2	23.1 (21.3-25.1)	23.1 (20.8-24.5)	0.467
Duration of disease, years	2 (0-14.5)	5 (1-16)	0.082
ESR, mm/hour	32 (19-46)	43 (25-60)	**0.024**
CRP, mg/dL	0.78 (0.33-1.84)	1.03 (0.37-2.53)	0.269
Baseline DAS28	4.6 (0.9)	5.0 (1.1)	**0.030**
RF, IU/mL	129.9 (28.3-484.6)	138.1 (47.8-528.6)	0.350
ACPA, U/mL	165 (32.3-340)	152 (35.2-340)	0.525
vdH Sharp score	8 (0-33)	17 (5-38.5)	0.090
Glucocorticoid use, n (%)	31 (58.5)	40 (81.6)	**0.011**
PD equivalent, mg/day	2.5 (0-5)	5 (2.5-5)	**0.019**
MTX dose, mg/week	7.5 (0-10)	10 (7.5-12.5)	**0.045**

Data are expressed as mean (standard deviation) or median (interquartile range) for continuous variables. c-DMARD: conventional disease-modifying anti-rheumatic drug; b-DMARD: biologic disease-modifying anti-rheumatic drug; BMI: body mass index; ESR: erythrocyte sedimentation rate; CRP: C-reactive protein; DAS28: disease activity score 28; RF: rheumatoid factor; ACPA: anti-citrullinated peptide antibodies; vdH: van der Heijde; PD: prednisone; and MTX: methotrexate.

Supplementary Table 4. Baseline characteristics of RA patients according to their treatment response to DMARD

Variable	Good or moderate response (n=82)	No response (n=21)	p value
Age, years	57.3 (11.0)	50.9 (12.8)	**0.022**
Female, n (%)	70 (85.4)	21 (100)	0.120
BMI, kg/m^2	23.2 (21.1-25.1)	22.3 (20.8-24.9)	0.466
Duration of disease, years	4 (0-15)	2 (0-13)	0.340
ESR, mm/hour	36 (19-52.3)	38 (23.5-50.5)	0.863
CRP, mg/dL	0.84 (0.34-2.09)	1.09 (0.36-1.86)	0.797
Baseline DAS28	4.9 (1.0)	4.5 (1.0)	0.087
RF, IU/mL	119.2 (37.9-499.8)	129.9 (49.5-405.1)	0.925
ACPA, U/mL	147 (28-340)	198.5 (141.9-340)	0.130
vdH Sharp score	10 (4-35)	12 (3-42)	0.937
Treatment			
Glucocorticoid use, n (%)	60 (73.2)	11 (52.4)	0.066
PD equivalent, mg/day	2.5 (0-5)	2.5 (0-5)	0.333
c-DMARD users, n (%)	40 (48.8)	13 (61.9)	0.283
b-DMARD users, n (%)	41 (50.0)	8 (38.1)	0.330
Baseline VEGF	0.028 (0.006-0.064)	0.028 (0.001-0.042)	0.519
Baseline IL-6	0.007 (0.003-0.018)	0.004 (0.002-0.016)	0.202
△VEGF (Baseline-F/U)	0.010 (0, 0.031)	-0.001 (-0.015, 0.016)	**0.011**
△IL-6 (Baseline-F/U)	0.003 (-0.003, 0.011)	0 (-0.005, 0.009)	0.494
△ESR (Baseline-F/U)	17 (9, 36.5)	6 (-3.5, 22)	**0.005**
△CRP (Baseline-F/U)	0.62 (0.10, 1.77)	0.61 (0.02, 1.17)	0.307

Data are expressed as mean (standard deviation) or median (interquartile range) for continuous variables. BMI: body mass index; ESR: erythrocyte sedimentation rate; CRP: C-reactive protein; DAS28: disease activity score 28; RF: rheumatoid factor; ACPA: anti-citrullinated peptide antibodies; vdH: van der Heijde; PD: prednisone; c-DMARD: conventional disease-modifying anti-rheumatic drug; b-DMARD: biologic disease-modifying anti-rheumatic drug; VEGF: vascular endothelial growth factor; IL-6: interleukin-6; and F/U: follow-up.
